# Survival and immune response of the Chagas vector *Meccus pallidipennis* (Hemiptera: Reduviidae) against two entomopathogenic fungi, *Metarhizium anisopliae* and *Isaria fumosorosea*

**DOI:** 10.1186/s13071-016-1453-1

**Published:** 2016-03-24

**Authors:** A. Laura Flores-Villegas, Margarita Cabrera-Bravo, Conchita Toriello, Martha I. Bucio-Torres, Paz María Salazar-Schettino, Alex Córdoba-Aguilar

**Affiliations:** Departamento de Microbiología y Parasitología, Facultad de Medicina, Universidad Nacional Autónoma de México, México, DF Mexico; Departamento de Ecología Evolutiva, Instituto de Ecología, Universidad Nacional Autónoma de México, Apdo. P. 70-275, Circuito Exterior, Ciudad Universitaria, 04510 Coyoacán, Distrito Federal Mexico

**Keywords:** Chagas disease, *Meccus pallidipennis*, Entomopathogenic fungi, Survival, Phenoloxidase, Prophenoloxidase

## Abstract

**Background:**

Chagas disease is a key health problem in Latin America and is caused and transmitted by *Trypanosoma cruzi* and triatomine bugs, respectively. Control of triatomines has largely relied on the use pyrethroids, which has proved to be ineffective in the long term. Alternatively, the use of entomopathogenic fungi has been implemented to control triatomine bugs. These fungi are highly efficient as they induce a reduction in immune response on insects. *Meccus pallidipennis* is the main triatomine vector of Chagas disease in Mexico. In this work we investigated the effects of two entomopathogenic fungi, *Metarhizium anisopliae* and *Isaria fumosorosea*, on *M. pallidipennis* nymphs in terms of insect survival and immune response.

**Methods:**

We had an infected and a control group for each fungal species and assessed: a) insect survival during 30 days; and, b) phenoloxidase (PO) and prophenoloxidase (proPO; two key traits in insect immune response) at 24, 48, 96 and 144 h. For survival we used Kaplan-Meier survival analysis while for immune response we used factorial, repeated-measures ANOVA for each fungal species.

**Results:**

Animals treated with *M. anisopliae* died sooner than animals treated with *I. fumosorosea*. Infected animals showed lower PO and proPO values than sham individuals, with a clear decrease in these parameters at 24 h with no further changes after this time.

**Conclusions:**

Our study widens the possibility of entomopathogenic fungi being used for triatomine control. The negative effect on PO and proPO seems mediated by a down-regulation of the triatomine immune response.

## Background

Chagas disease is caused by the protozoan *Trypanosoma cruzi* and is considered endemic from Mexico to Argentina [1]. The main transmission source is vectorial, through insect bugs of the subfamily Triatominae (Hemiptera: Reduviidae) [2]. For the case of Mexico, *Meccus pallidipennis* is responsible of approximately 74 % of vectorial transmission (Martínez-Ibarra et al. [3]). The biology of *M. pallidipennis* explains such high transmission rate: a) a wide distribution in Mexico [4, 5] b) its relatively high abundance compared to other triatomines [6] c) its peridomestic nature, meaning close contact with humans [6] d) it is one of the triatomines with the highest *T. cruzi* infection rates [7]; and e) its capacity as highly efficient vector in terms of egg incubation and hatching time, egg-to-adult mortality, oviposition rate and post-feeding defecation times [8].

Given the risk that *M. pallidipennis* implies for *T. cruzi* transmission, there have been several efforts to control it. One of these is the use of synthetic pyrethroids such as bifenthrin, cyfluthrin and deltamethrin insecticides. However, these pyrethroids are not reliable given the high bug re-infestation in dwellings after pyrethroid spraying [9, 10], which is associated with insecticide resistance [11, 12]. Although some other control methodologies have been proposed and/or explored (e.g. using genetically-modified endosymbiont bacteria that impede *T. cruzi* development [13], using natural predators [14]), possibly more than one control action is needed [15]. Here we explored the use of two entomopathogenic fungi, *Metarhizium anisopliae* (Hypocreales: Clavicipitaceae) and *Isaria fumosorosea* (Hypocreales: Cordycipitaceae)*,* against *M. pallidipennis* nymphs. Use of these fungi has been highly successful for controlling different insect pests and vectors such as Asian tiger mosquitoes *Aedes albopictus* [16], cattle ticks *Rhipicephalus microplus* [17], Asian citrus psyllids *Diaphorina citri* [18] and diamondback moth *Plutella xylostella* [19]. As a matter of fact, several strains of *M. anisopliae* and *I. fumosorosea*, that are highly efficient to kill triatomines, have been identified [20–23]. Thus, entomopathogenic fungi seem a viable route for triatomine biological control [12, 23–25].

The mechanism that makes entomopathogenic fungi so successful during insect attack, starts with the conidium contact with the insect cuticle [26]. During this, the fungus adheres, penetrates, disseminates and exits the insect body [27]. Once inside, the fungus evades the insect’s immune system by: 1) enzyme (e.g. proteases and chitinases) development that degrades the insect cuticle; 2) development of blastospores and hyphal bodies in the hemolymph which inhibit insect immune response; and 3) production of secondary toxic metabolites such as destruxins and beauvericins [28]*.* However, perhaps the largest attribute of entomopathogenic fungi as a vector and pest control relies on their immunosuppressive action. In support of these, several studies have found that two key insect immune players, phenoloxidase (PO) and, its precursor, prophenoloxidase (proPO), become down-regulated during fungal infection in some, but not all, insect species [29–31]. It is known that during the course of infection, insects make use of PO and proPO against a plethora of pathogens [32, 33]. The enzymatic process from proPO to PO is regulated by a complex proteolytic cascade, which is activated by the recognition of cell wall components of fungi and other pathogens [32]. Furthermore, PO gives rise to cuticle sclerotization and wound repair but also promotes melanine biosynthesis during the formation of nodules and encapsulation of pathogens [34, 35]*.* Although it is unclear how PO and proPO activity is inhibited, one hypothesis is that fungal destruxins destroy those proteins present in insect cells responsible for proPO production [36, 37].

The main aim of this study was to test the efficiency of two entomopathogenic fungi, *Metarhizium anisopliae* and *Isaria fumosorosea*, to control *M. pallidipennis* nymphs. For this, first we infected bugs using each fungal species individually and assessed survival. Then, to understand the physiological mechanism underlying fungal infection, we recorded the activity of PO and proPO using repeated time measures of the same individual.

## Methods

### Insects

We used 5th stage nymphs of *M. pallidipennis,* from a colony maintained in the insectary of the Biology of Parasites, Microbiology and Parasitology Department, Faculty of Medicine, Universidad Nacional Autónoma de México. This colony was established in 1998 from insect individuals collected from Oaxtepec village (18°54′23″N, 98°58′13″W), state of Morelos, Mexico. Insects were maintained under controlled conditions of 60 % relative humidity, 28 °C, and 12/12 h light/dark cycles at the laboratory.

### Nymph infection

#### Fungi

We used monosporic cultures of *M. anisopliae* EH-473/4 and *I. fumosorosea* EH-511/3 strains whose insect virulence, pheno-and genotypic characterization and safety for mammals are well known [38–40]. These fungi are part of the culture Collection of the Basic Mycology Laboratory, Microbiology and Parasitology Department, Faculty of Medicine, Universidad Nacional Autónoma de México, registered in the “World Federation of Culture Collections” (WFCC) as BMFM-UNAM 834. The original fungal strains were obtained from the “Colección de Hongos Entomopatógenos (CHE)” from the Centro Nacional de Referencia de Control Biológico (CNRCB), Colima City, Mexico. *M. anisopliae* was isolated in 1994, from *Aeneolamia* sp. (Hemiptera: Cercopidae), from a sugarcane crop in San Luis Potosi city, Mexico. The label of *M. anisopliae* at CNRCB is CHE-CNRCB 227. The other fungus, *I. fumosorosea* was isolated in 1994 from *Bemisia* sp. (Hemiptera: Aleyrodidae) from a watermelon crop in Colima, Mexico. The label of *I. fumosorosea* at CNRCB is CHE-CNRCB 304. Fungi were previously cultivated in potato dextrose agar (PDA, g/l: 300 g of white potato, 20 g of dextrose, 15 g of agar (BIOXON®, México) and then incubated at 28 °C for eight days [41].

*Conidial suspension.* The conidia were produced in PDA medium cultures and incubated at 28 °C for 7 days. After incubation, conidia were obtained using 3 ml of 0.5 % Tween 80. This suspension was kept on ice throughout the bioassay, homogenized and two dilutions were performed: 1:10 and 1:100. The number of conidia was counted in a Neubauer chamber and the suspension was adjusted to obtain a final concentration of 1 × 10^7^ conidia/ml for the infection procedure. The whole procedure was performed in a laminar flow hood [42].

### Survival assessment

#### Infection procedure and infected group

We applied 30 μl from a suspension of 1 × 10^7^ conidia/ml of *M. anisopliae* or *I. fumosorosea* on each nymph’s pronotum. Each nymph was placed individually in a sterile plastic Petri dish with sterile filter paper and was incubated at 28 °C with a 12/12 h light/dark cycle. After 24 h, all infected nymphs were transferred to 1 % agar-water (to provide appropriate humidity conditions for fungal growth) in plastic Petri dishes (100 × 15 mm) and were again incubated using a Precision 818® incubator at 28 °C and 80 % relative humidity for one month. We corroborated that the infection took place by assessing presence of hyphae and/or mycelium on the insect, and we recorded the number of dead insects daily [40, 42, 43]. We had three criteria to assess that an insect was dead due to fungal infection: a) signs of mycelium presence on the cuticle (by direct observation under a stereoscopic microscope, Olympus SZ40); b) presence of fungal structures inside the insect using imprints on the day when dead insects were recorded; and c) motionless insects. Furthermore, we took fungal samples from nine infected insects of each fungus. This was done by taking some sporulating fungi emerging from the insect cuticle, using an inoculating loop under a flow hood. Fungal samples were cultivated in PDA cultures, to corroborate the micro- and macroscopic characteristics of each infecting fungal species. For imprints, the insect cuticle surface was cleaned using 70 % ethanol and 40 % sodium hypochlorite to remove potential contaminants [42]. Subsequently, a longitudinal (from head to posterior end) cut was gently made using scissors and the cuticle was removed. A drop of blue cotton was then placed on the internal area of the cuticle, and this structure was placed on a slide, covered with a coverslip and sealed with nail varnish to be observed under a microscope at 40×. Each evaluated group had 10 insects with 5 replicates for each fungus, i.e. 50 insects per fungal strain infection.

#### Sham group

We applied 30 μl of 0.5 % Tween 80 on each nymph’s pronotum. Each animal was then moved to a Plastic Petri dish with sterile filter paper and was incubated at 28 °C with a 12/12 h light/dark cycle. Except for the fungal infections, this sham group was treated under the same conditions as the infected groups: 24 h after applying 30 μl of 0.5 % Tween 80, all insects were transferred to agar-water (1 %; again to provide humidity conditions for fungal growth) in plastic petri dishes (100 × 15 mm) (see [43]) and were incubated using a Precision 818® equipment at 28 °C and 80 % relative humidity for one month. Similar to the infected group, mortality of sham insects was recorded daily for one month. To assess whether an insect was dead, we also applied the three criteria expressed above. Similar to the infected groups, we used 10 insects with 5 replicates for each fungus, i. e. 50 insects for each fungal strain.

### Negative effects of fungal infection on PO and proPO

We used the same rationale of infection and incubation for those animals described above for the survival experiment. However, rather than assessing survival, we collected hemolymph from both groups every 24, 48, 96 and 144 h after the infection for both fungal species.

#### Hemolymph extraction

Using a 1 ml micro syringe, we punctured the insect membrane that separates the coxa and trochanter of one of the posterior legs [44], and gently pushed the abdomen. The emerging hemolymph was then collected with a 10 μl micropipette. Hemolymph was mixed with PBS pH 7.2 1 × 2.9 g of Na_2_HPO_4_.12H_2_O, 0.2 g of KH_2_PO_4_, 0.2 g of KCl, 8.0 g of NaCl and deionized water, in a proportion of 1:2.

#### Protein concentration

For protein quantification and standardization in our samples, we used the Pierce method with the BCA commercial kit (Thermo Fisher Scientific, Rockford, Illinois). For this, in each of 96 microwells of a plate (Costar 96; Corning, New York, New York) we placed 10 μl of hemolymph, 40 μl of PBS pH 7.2 1 X and 150 μl of the Pierce re-agent. We used 2 mg/ml albumin to obtain a standard curve. The plate was covered with foil and was incubated at 37 °C for 30 min. Absorbance was measured in an ELISA plate reader (ELX 800, Biotek) at 562 nm. Protein content was adjusted to 10 μg of protein to record PO and proPO activity [45].

#### PO activity

PO activity levels were quantified spectrophotometrically through catalytic conversion of L-dopa 3, 4-dihydroxi-L-phenylalanine (colorless) to dopachrome (brown-red) [46]. Again using a 96 microwell plate, we added 10 μg of protein of each sample contained in 100 μl of PBS. To start PO activation, we added 100 μl of the L-DOPA substrate to a 4 mg/ml concentration. The plate was incubated at 37 °C for 20 min in the dark. After this 20-min period, readings were taken with an ELISA plate reader (ELX 800, Biotek) at 490 nm each 5 min for one hour (giving a total of 12 readings). As blanks we used 100 μl of PBS with 100 μl of L-Dopa. PO readings were obtained in different time periods: after 24, 48, 96 and 144 h. PO activity was expressed as enzyme units (U), where 1 U is the enzyme amount which produces 1 μmol of dopachrome (product) per minute [47].

#### proPO activity

proPO activity was recorded *via* an artificial activation with α-chymotrypsin [46]. We used 1 μg/ml of α-chymotrypsin (Sigma®). Using the same 96 microwell plate described above, we added 45 μl of PBS, 20 μl of hemolymph sample and 5 μl of α- chymotrypsin. This mixture was incubated at 37 °C for 20 min in the dark. After this 20-min period, 130 μl of L-Dopa was added, and the resulting readings were recorded at 490 nm, each 5 min for one hour (giving a total of 12 readings). As blanks, we used 65 μl of PBS with 130 μl of L-Dopa. proPO readings were obtained in different time periods: after 24, 48, 96 and 144 h. To record specific proPO activity, we did the same as for PO described above.

### Statistical analyses

We used Kaplan-Meier analysis for survival. Average survival time as well as risk ratio index were analyzed using a Log-rank (Mantel-Cox) test. For analyzing the effect of treatment on PO and proPO, we used four factorial, repeated-measures ANOVA for each fungal species, with time of PO and proPO assessment as the repeated measure and treatment (experimental, sham) as the between-group variable. Previously, and to fulfill normal distribution assumptions of PO and proPO, we log transformed our data. Analyses were carried out with the repeated measures module of SPSS version 21.

## Results

### Survival

**Fig. 1:**
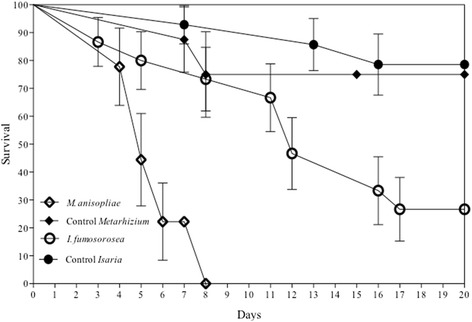
Survival of fifth instar nymphs of *Mccus pallidipennis* infected with *M. anisopliae* (EH- 473/4), *I. fumosorosea* (EH- 511/3) and their control groups

There were differences in survival distribution for the four treatments (*X*^2^_(3)_ = 29.12, *P* < 0.0001; Fig. 1). A comparison of both fungal treatments indicated that individuals treated with *M. anisopliae* died sooner than those treated with *I. fumosorosea* (*X*^2^_(1)_ = 11.49, *P* = 0.0007). For those treated with *I. fumosorosea* as well as the control groups of both fungi, no further mortality was detected after 20 days.

**Fig. 2:**
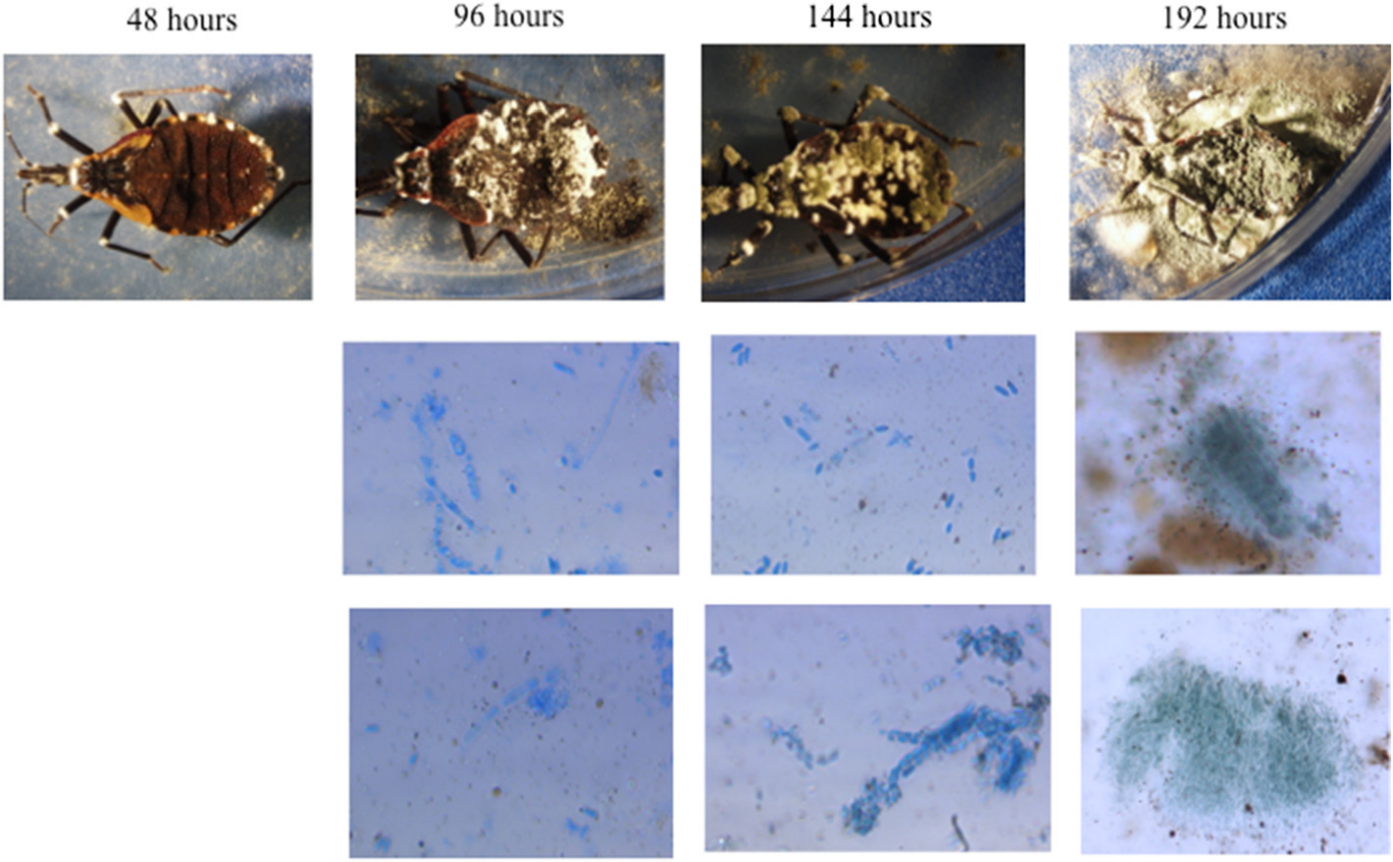
Fifth instar nymphs of *M. pallidipennis* infected with *M. anisopliae* (EH- 473/4; first row of pictures) and presence of fungal structures (second and third row) on the internal area of the cuticle along time. These fungal structures are shown as hyphal bodies, conidia and conidia columns (40×)

**Fig. 3:**
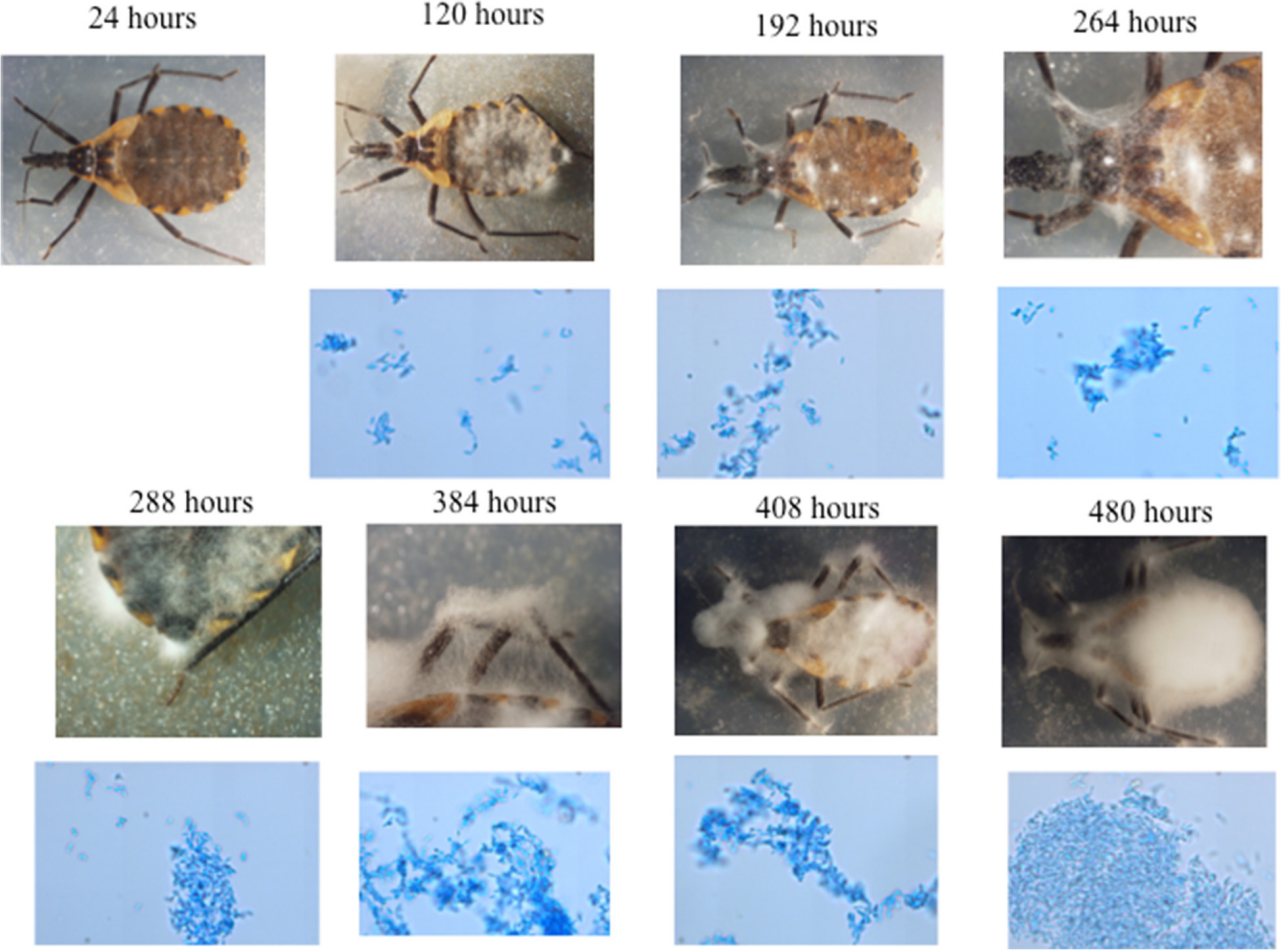
Fifth instar nymphs of *M. pallidipennis* infected with *I. fumosorosea* (EH- 511/3; first and second row) and presence of conidia (40×; second and fourth row) along time

**Fig. 4:**
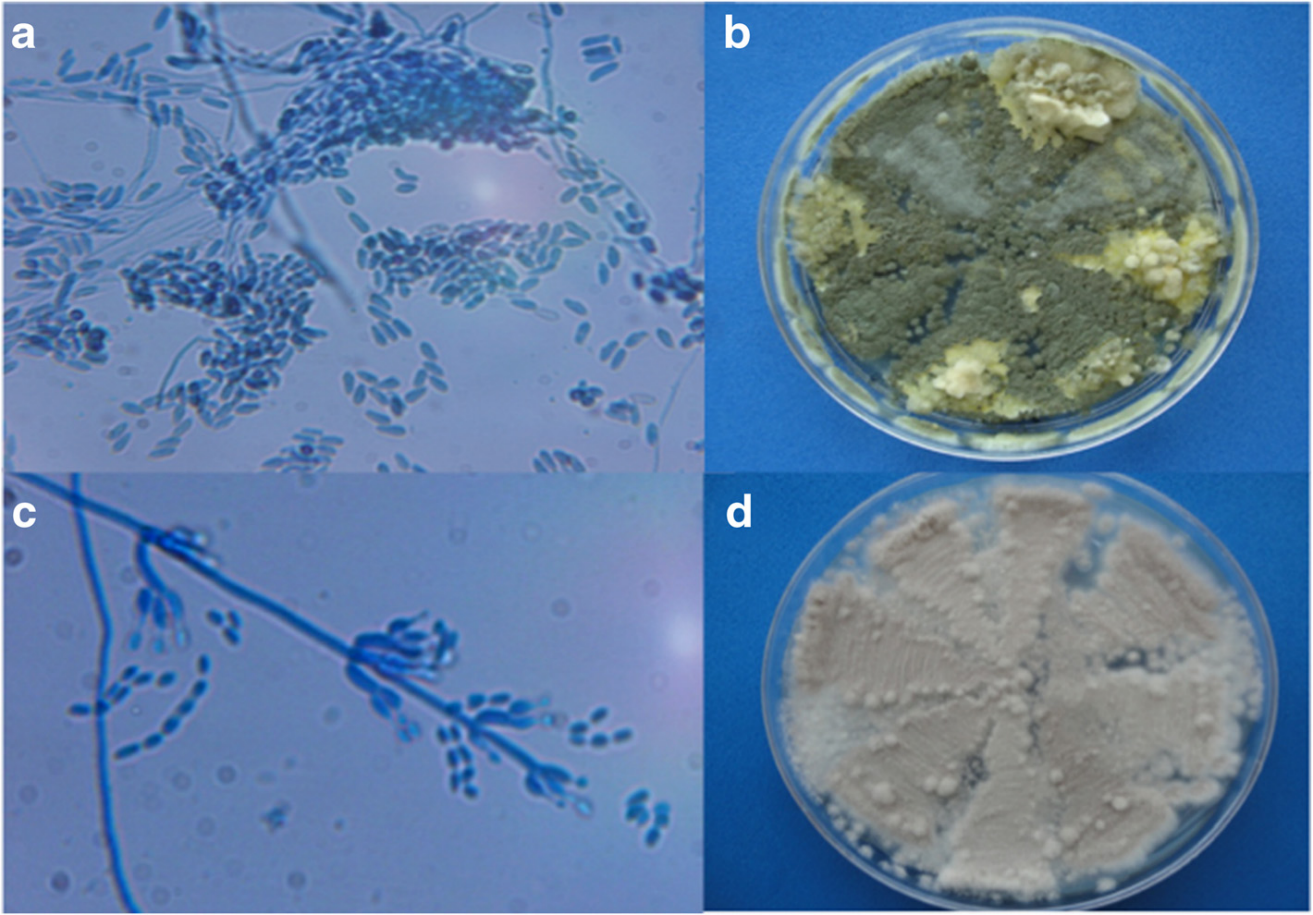
Microscopic and macroscopic features after fungal growth of samples recovered from nine nymphs infected with *M. anisopliae* (EH- 473/4; **a**, **b**) and *Isaria fumosorosea* (EH- 511/3; **c**, **d**)

Imprints indicate a large invasion of both fungal species within triatomines (Figs. 2 and 3). In relation to assessment of fungal infections, cultures from the nine infected nymphs with *M. anisopliae* showed the following microscopic features: branched conidiophores with basipetal catenulate cylindrical conidia (Fig. 4a). In regards to macroscopical characteristics, initially the colony had a white color that, after several days, turned into an olive green color (Fig. 4b). Cultures from the nine infected nymphs with *I. fumosorosea* showed microscopic fungal features such as simple conidiophores, with a globose basal portion with emerging fusiform conidia (Fig. 4c). As for macroscopic characteristics, a typical white cotton-like colony was observed with gray-pinkish color when sporulated (Fig. 4d).

### PO and proPO activity according to fungal treatment

#### Metarhizium anisopliae *infection*

**Fig. 5:**
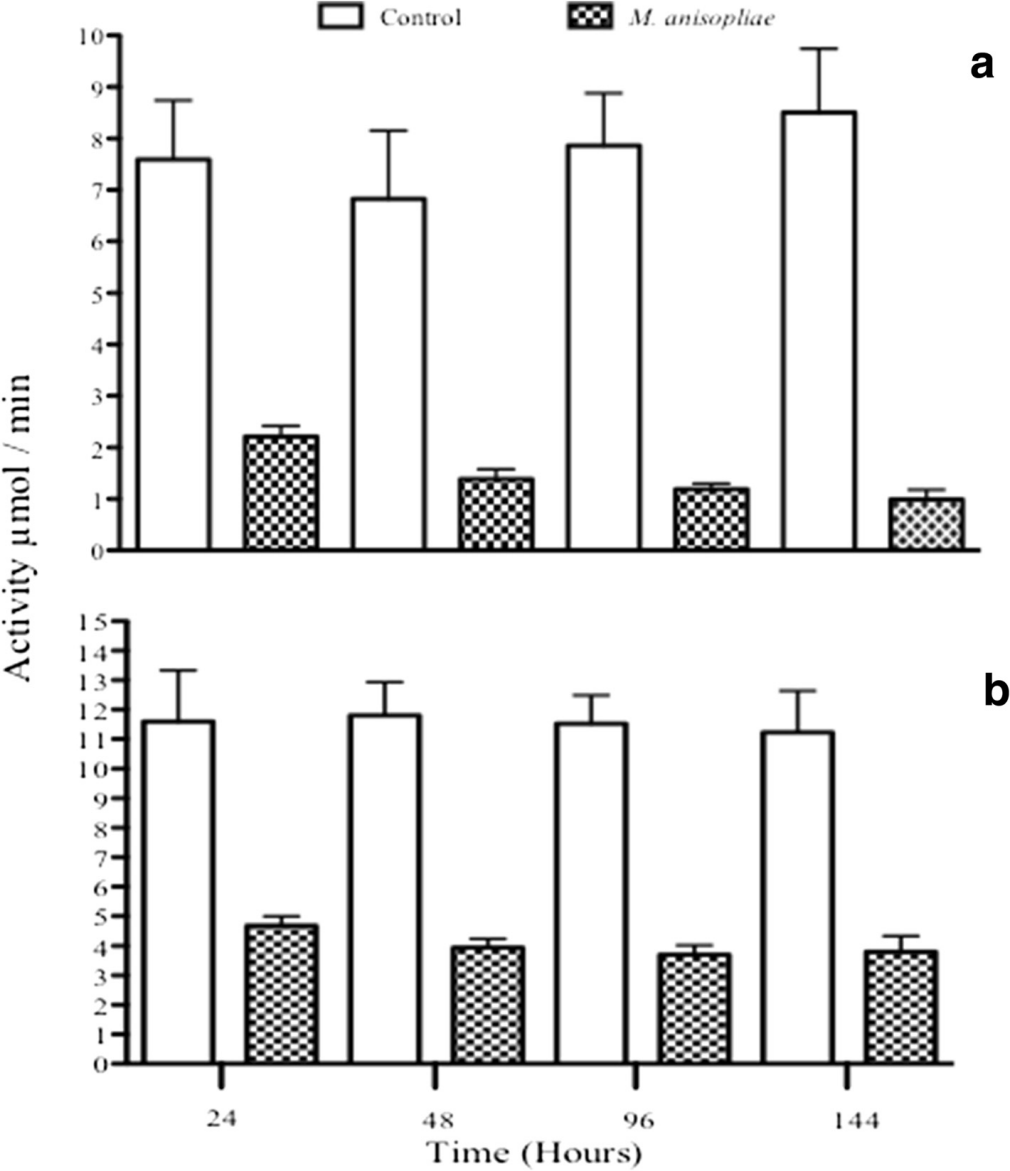
PO (**a**) and proPO (**b**) activity responses in fifth instar nymphs of *M. pallidipennis* infected with *M. anisopliae* (EH- 473/4) along time

The general model for predicting PO changes indicated that the interaction time*treatment was significant (*F*_3,37_ = 11.721, *P* < 0.0001). Changes in PO were not significant along time (*F*_3,37_ = 0.692, *P* = 0.563) but treatment was (*F*_1,39_ = 69.035, *P* < 0.0001). According to the latter, PO showed lower values in the infected group (Fig. 5a).

The general model for proPO changes was not significant (*F*_3,32_ = 2.192, *P* = 0.108) with non-significant differences along time (*F*_3,32_ = 0.144, *P* = 0.932) but there was a clear negative effect according to treatment (*F*_1,34_ = 31.737, *P* < 0.0001) where the infected insects ended up with lower values than sham insects (Fig. 5b).

#### Isaria fumosorosea *infection*

**Fig. 6:**
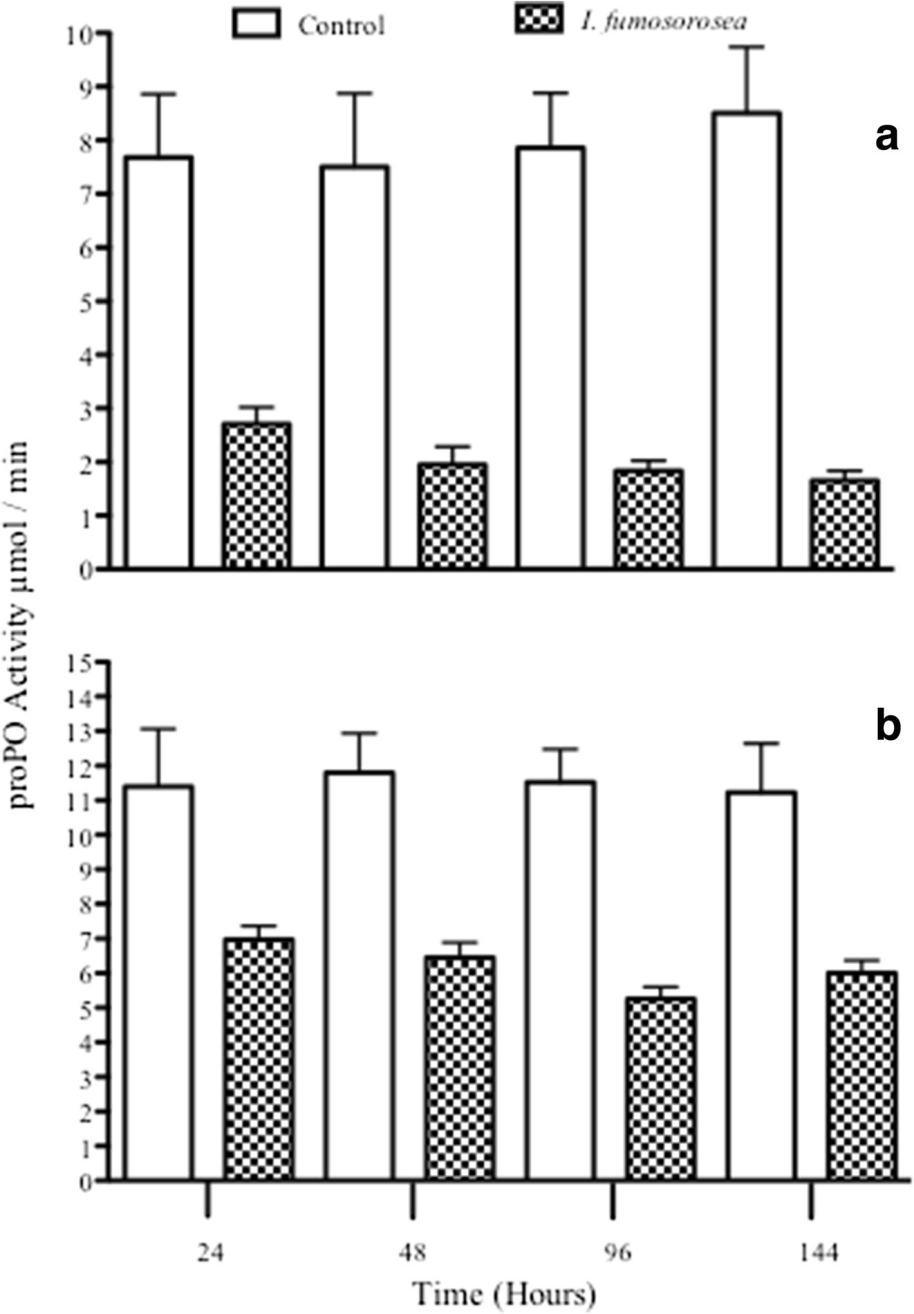
PO (**a**) and proPO (**b**) activity responses in fifth instar nymphs of *M. pallidipennis* infected with *I. fumosorosea* (EH-511/3) along time

There was a significant change in PO when the general model for the interaction time*treatment was examined (*F*_3,31_ = 4.812, *P* = 0.007). Time did not predict PO changes (*F*_3,31_ = 0.522, *P* = 0.671) but treatment did, resulting in lower values for infected insects (*F*_1,33_ = 61.072, *P* < 0.0001; Fig. 6a).

ProPO did not show significant changes according to the interaction time*treatment (*F*_3,21_ = 2.337, *P* = 0.103). Time did not affect proPO (*F*_3,21_ = 0.662, *P* = 0.584) but treatment did with lower values for infected insects (*F*_1,23_ = 28.543, *P* < 0.0001; Fig 6b).

## Discussion

Our survival experiment indicated differences in killing *M. pallidipennis*, with *M. anisopliae* with a higher virulence than *I. fumosorosea*. As a matter of fact, the former fungus killed all animals at day 8 while the latter took longer than 30 days. These differences in the action of both fungi are likely due to a stronger effect of *M. anisopliae* when compared to *I. fumosorosea.* For example, *M. anisopliae* produces immunosuppressor toxins like destruxin [48] that may lead to damage hemocytes [49] and cause insect paralysis (*via* an increased calcium level in Malpighian tubules) [50]. These effects take place possibly due to the ability of *M. anisopliae* to produce a collagenous coat of hyphal bodies that mask the recognition of β-1,3-glucans by the insect immune system [51]. In contrast, little is known in regards to the effect of *I. fumosorosea*. It is clear that this fungus takes longer to sporulate than *M. anisopliae,* possibly due to a more paused beauvericine production [52] and hyphal growth [43]. How such effects take place *via* inhibition of the insect immune response, is unclear. Our results partly echo findings in other triatomines in which *M. anisopliae* was used [23, 53, 54]. To our knowledge, however, *I. fumosorosea* has been less extensively used against triatomines and one response may be that, as we have documented, *M. anisopliae* seems more effective. In fact, other studies in non-triatomines have corroborated that *M. anisopliae* is more effective than *I. fumosorosea* [55–57]. It would be interesting to compare our results with those occurring at other triatomine ages. Unfortunately, such studies have not been carried out yet.

Overall, we found that after fungal infection, both proPO and PO activity decreased. A first defense line in insects is that of the cuticle, where fungistatic fatty acids, phenoloxidases and melanins impede fungal penetration [58]. In case such barrier is overcome, *via* degradation of insect cuticle hydrocarbons [59], then the insect immune system makes use of several humoral and cellular components that include hemocytes that encapsulate fungal parts, PO, reactive oxygen species and antimicrobial peptides [60]. For the case of PO, this synthesizes melanin whose antifungal activity acts directly on the fungal surface which stops fungal development [36, 61]. Despite this role for PO, and its precursor, proPO, these immune components have been shown to decrease during fungal infection [29–31] although the underlying mechanism for such decrease is unclear. In relation to this, fungal infection using *Beauveria bassiana* in the migratory grasshopper *Melanoplus sanguinipes* [62] and of *M. anisopliae* in the locust *Schistocerca gregaria* [29, 63], led to a reduction in hemocyte number. One way this negative action can occur is *via* the use of fungal mycotoxins such as destruxin [48]. This compound reduces PO activity, phagocytosis and encapsulation [52]. It is unclear, however, whether such reduction accompanies or is also a function of a reduction in hemocyte number. One reason why we should expect a relation among these negative actions is that hemocytes carry out the PO cascade so that if these cells are affected by fungus, possibly in the form of lysis [64], proPO and PO are affected too. Furthermore, looking more closely at proPO values after infection by both fungi, this immune response showed higher activity after *I. fumosorosea* than after *M. anisopliae* treatment (Figs. 5 and 6). One has to remember that proPO is the resource tool for PO production [32] so that if both fungi had the same inhibitory PO response, proPO values should remain the same after the infection by either pathogen. Given the hypothesis of an inhibitory response by our fungi, the insect still makes use of some proPO for PO production after *M. anisopliae* treatment (or at least more so than after *I. fumosorosea* infection). This would imply that the insect shows some PO-based immune response against *M. anisopliae* so that the inhibitory response by this fungus is not as complete (or less complete) than that elicited by *I. fumosorosea* infection. This hypothesis would need further testing.

Given a first decrease in PO and proPO at 24 h, we did not detect further changes in these immune components. Other studies in insects have found an initial increase in PO after 10 min of infection [64], followed by a general decrease at 24 h and no clear changes after this time [29, 64]. This implied an activation of an immune response followed by a negative effect of fungi on the entire PO cascade. Although we did not check what occurred soon after fungal infection, our results confirm previous claims that fungal infection affects PO and proPO [29–31].

Given that there are no vaccines available for controlling Chagas disease, its control relies on local preventive measures which, historically, has been based on the use of insecticides. One alternative to this chemical control is the use of entomopathogenic fungi which has been implemented in some countries [12] but not in Mexico. Our findings indeed seem promising although our conditions still prevent further implementation. For these conditions we refer, for example, to the fact that we used triatomine nymphs. It is known that effects of entomopathogenic fungi on insects may vary according to the ontogenetic insect stage used [65]. This means, that the effect of other stages in *M. pallidipennis* needs to be evaluated. On the other hand, perhaps the use of more than a single strategy is needed to control triatomines [15, 33]. One way to do this is using different pathogens to which insects, or more specifically triatomines, are susceptible. Such pathogens are, for example, the bacteria *Serratia marcescens* [66, 67] Triatoma virus [68, 69] or, even, other fungi [33].

## Conclusions

As opposed to the use of pyrethroids, entomopathogenic fungi can be used to control triatomine bugs, the vectors of Chagas disease. We showed that *M. anisopliae* fungus is actually a better control tool than *I. fumosorosea* for killing 5th stage nymphs of *M. pallidipennis*. Our results provide some light to such killing mechanism as, along with fungal infection, we detected a reduction in PO and proPO enzymes, two key components in insect immune defense. Possibly, fungi compromise immune ability *via* mycotoxins such as dextrusin as has been shown in other studies. Thus, our results imply an alternative tool for Chagas disease control.
